# Rare and common variants in *ROM1* and *PRPH2* genes *trans*-modify Stargardt/ABCA4 disease

**DOI:** 10.1371/journal.pgen.1010129

**Published:** 2022-03-30

**Authors:** Jana Zernant, Winston Lee, Jun Wang, Kerry Goetz, Ehsan Ullah, Takayuki Nagasaki, Pei-Yin Su, Gerald A. Fishman, Stephen H. Tsang, Santa J. Tumminia, Brian P. Brooks, Robert B. Hufnagel, Rui Chen, Rando Allikmets

**Affiliations:** 1 Department of Ophthalmology, Columbia University, New York, New York, United States of America; 2 Department of Genetics & Development, Columbia University, New York, New York, United States of America; 3 Department of Molecular and Human Genetics, Baylor College of Medicine, Houston, Texas, United States of America; 4 National Eye Institute, NIH, Bethesda, Maryland, United States of America; 5 The Pangere Center for Inherited Retinal Diseases, The Chicago Lighthouse, Chicago, Illinois, United States of America; 6 Department of Pathology & Cell Biology, Columbia University, New York, New York, United States of America; University of Texas Health Science Center, UNITED STATES

## Abstract

Over 1,500 variants in the *ABCA4* locus cause phenotypes ranging from severe, early-onset retinal degeneration to very late-onset maculopathies. The resulting ABCA4/Stargardt disease is the most prevalent Mendelian eye disorder, although its underlying clinical heterogeneity, including penetrance of many alleles, are not well-understood. We hypothesized that a share of this complexity is explained by *trans*-modifiers, i.e., variants in unlinked loci, which are currently unknown. We sought to identify these by performing exome sequencing in a large cohort for a rare disease of 622 cases and compared variation in seven genes known to clinically phenocopy ABCA4 disease to cohorts of ethnically matched controls. We identified a significant enrichment of variants in 2 out of the 7 genes. Moderately rare, likely functional, variants, at the minor allele frequency (MAF) <0.005 and CADD>25, were enriched in *ROM1*, where 1.3% of 622 patients harbored a *ROM1* variant compared to 0.3% of 10,865 controls (p = 2.41E04; OR 3.81 95% CI [1.77; 8.22]). More importantly, analysis of common variants (MAF>0.1) identified a frequent haplotype in *PRPH2*, tagged by the p.Asp338 variant with MAF = 0.21 in the matched general population that was significantly increased in the patient cohort, MAF 0.25, p = 0.0014. Significant differences were also observed between ABCA4 disease subgroups. In the late-onset subgroup, defined by the hypomorphic p.Asn1868Ile variant and including c.4253+43G>A, the allele frequency for the *PRPH2* p.Asp338 variant was 0.15 vs 0.27 in the remaining cohort, p = 0.00057. Known functional data allowed suggesting a mechanism by which the *PRPH2* haplotype influences the ABCA4 disease penetrance. These associations were replicated in an independent cohort of 408 patients. The association was highly statistically significant in the combined cohorts of 1,030 cases, p = 4.00E-05 for all patients and p = 0.00014 for the hypomorph subgroup, suggesting a substantial *trans*-modifying role in ABCA4 disease for both rare and common variants in two unlinked loci.

## Author summary

Although recent studies have deciphered most of the genetic variation underlying ABCA4/Stargardt disease, including non-coding variants and *cis*-modifiers in the *ABCA4* locus, many causal and modifying variants are still unknown. Here, we take advantage of recent advances in genetic and clinical methodology, and large for a Mendelian disease fully characterized patient cohorts, to examine the role of rare and common genetic variants in unlinked loci; i.e., *trans*-modifiers, for *ABCA4*-associated phenotypes. By examining 622 patients with ABCA4 disease, we show that rare variants in the *ROM1* gene and a common haplotype in the *PRPH2* gene are associated with ABCA4 disease overall, and with a specific disease subgroup. We replicated these findings in an independent cohort of 408 patients and suggest a mechanism for how the unlinked variation may functionally influence the penetrance of ABCA4 disease. These associations are true for populations of mostly European descent and suggest *trans*-modifying events in ABCA4/Stargardt disease by variation in other, unlinked, genes. They also serve as an example for other, considered “simple monogenic”, Mendelian diseases where careful consideration should be given to both rare and common variation in unlinked loci.

## Introduction

The concept of genetic modifiers, i.e., the role of genetic background in penetrance and expressivity of causal variants in Mendelian diseases, has recently gained substantial momentum. This is due to two factors; first technological advances since the advent of next-generation sequencing have resulted in expanding knowledge of causal genes and large cohorts of genetically characterized cases. For diseases of the retina, the progress is catalogued in RetNet where, as of January 2021, >270 genes, variants in which cause simple or syndromic retinopathies are currently listed (https://sph.uth.edu/retnet/disease.htm). Second, for retinal diseases similar advances have occurred in clinical characterization due to vastly improved imaging technologies, allowing for an extremely detailed subcategorization of Mendelian disease patients. The combination of genetic and clinical advances allows precise subcategorization of patients with comprehensive genetic data thereby significantly increasing the power of statistical analyses for identifying modifiers.

Despite these advances, the knowledge of genetic modifiers in retinal diseases have remained modest, with the exception of a group of diseases called ciliopathies, where numerous cases of *cis*- and *trans*-modifiers have been identified [1,2]. In this case the research has been aided by the fact that all ciliopathy genes form, or are associated with, the ciliary complex where they interact functionally (allelic interactions). Examples of allelic heterogeneity in retinal diseases are groups of genes causing disease entities called “retinitis pigmentosa” or achromatopsia, where the search for modifiers takes advantage of the fact that variants causing an overlapping Mendelian phenotype can modify the severity of disease expression [3–7].

The situation is more complicated for monogenic diseases where extensive allelic heterogeneity is not supported by knowledge of allelic interactions. The prime example for this situation are phenotypes caused by variants in the ATP-binding cassette transporter ABCA4 [8]. Variation in *ABCA4* causes extensive clinical heterogeneity, from early-onset, severe disease, rapid-onset chorioretinopathy, to very late-onset mild phenotypes that can be misdiagnosed as age-related macular degeneration (AMD) [9–12]. While, in most cases, the clinical heterogeneity is explained by genetic variation in the coding sequences of the *ABCA4* gene, several examples of *cis*-modifiers, that is variants in the *ABCA4* locus, are known [13–15]. These variants, often relatively frequent in the general population, affect penetrance and/or disease expression/progression [16]. Until now variants in other genes, i.e., *trans*-modifiers, have not been implicated as influencing ABCA4 disease. However, the existence of *trans*-modifiers is suggested by a number of observations. While there is no evidence that ABCA4 forms a functional complex with other proteins as in the ciliopathies, the central pathology in ABCA4 disease, lipofuscin formation and toxicity, is also found in other diseases (paracentral retina degeneration, flecked diseases, etc.) [17–19]. Furthermore, ABCA4 is in close proximity to proteins that have a shared functional ontology related to photoreceptor disc morphogenesis, structure, and integrity, e.g., PROM1, CDHR1, PRPH2, ROM1, etc. Compromised function of these proteins results in phenotypes mimicking ABCA4 disease. For example, we showed recently that PROM1 disease can be exacerbated by a deleterious *ABCA4* mutation [20] and it has been shown that ABCA4 is downregulated in Prom1^-/-^ mice [21]. However, the functional autonomy of ABCA4, unlike the described cases of causal genes in ciliopathies, achromatopsia and RP, suggests that any significant *trans*-modifying effect would likely be exerted indirectly to pathophysiology of ABCA4 disease and have measurable influence at population scale, rather than in any given affected individual.

Variants in a subset of retinal disease genes cause disease phenotypes overlapping with features of ABCA4 disease [8]. In a cohort of 145 patients with STGD1-like phenotypes, negative for mutations in *ABCA4* gene, we identified pathogenic variants in *PRPH2* and *PROM1* in ~20% of cases for both genes, and in *CRX* in 6% of cases [22]. Additionally, less frequently mutated genes causing phenotypes resembling ABCA4 disease include *ROM1*, *CDHR1*, *CHM*, and *ELOVL4* [22,23].

Therefore, we tested a hypothesis that rare or common variants in these seven genes, *CDHR1*, *CHM*, *CRX*, *ELOVL4*, *PROM1*, *PRPH2* and *ROM1*, known to cause monogenic retinal diseases with overlapping phenotypes with STGD1/ABCA4 disease [8,22,23], can also act as modifiers for the disease in general, or for genetic/phenotypic subgroups. We limited the analyses to these seven macular dystrophy genes from both clinical evidence and to increase the statistical power of the analyses. The analyses revealed enrichment of both rare (MAF<0.005), presumably highly penetrant, and common (MAF~0.1–0.2) variants in two of the seven genes (*ROM1* and *PRPH2*) in STGD1/ABCA4 disease. The strongest association was with a common haplotype in the *PRPH2* gene with both specific *ABCA4* genotypes and the entire ABCA4/STGD1 disease.

## Results

### Study design

**Fig 1:**
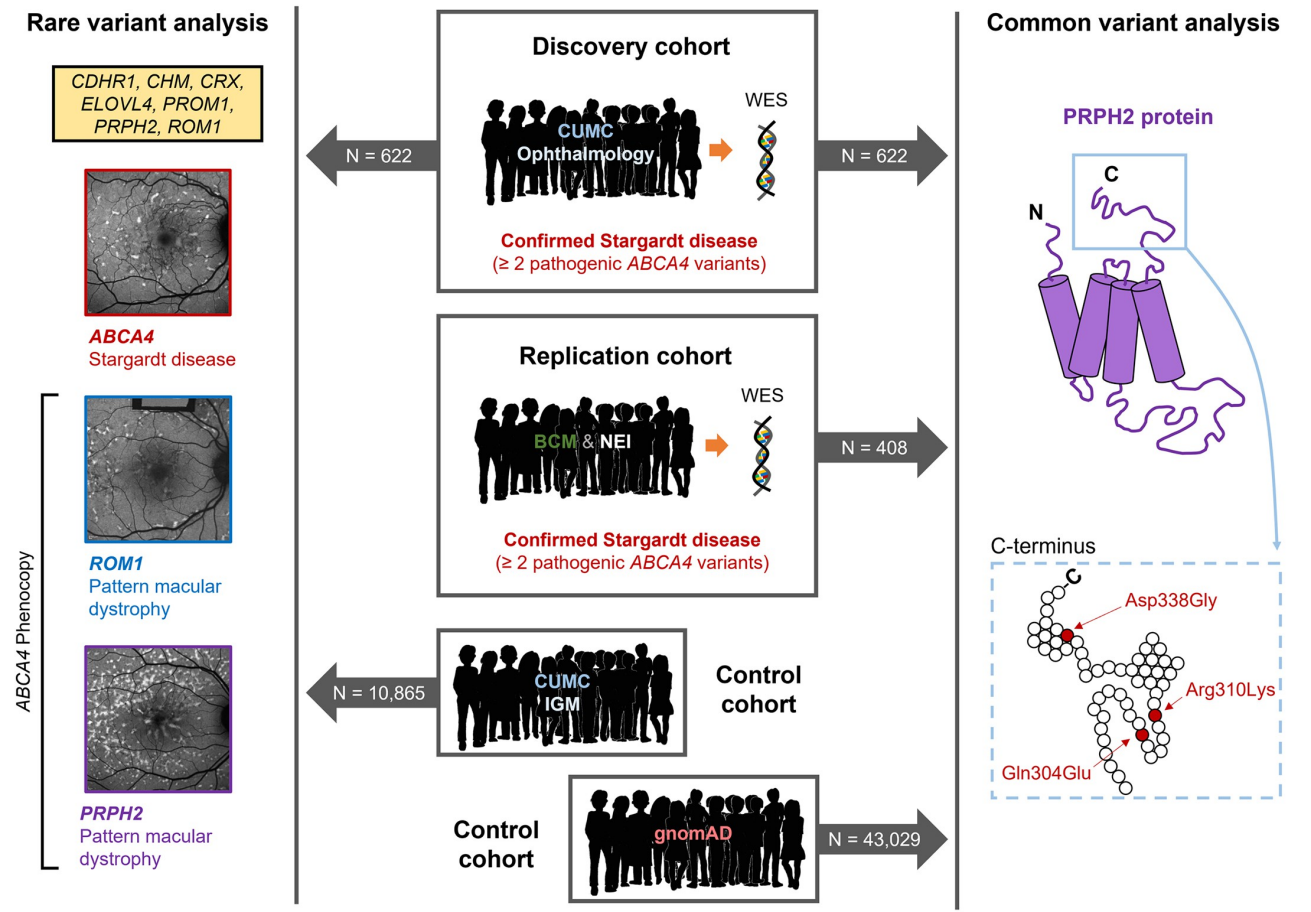
Study design and methodology used to identify *trans*-modifiers in ABCA4 disease. Rare and predicted deleterious variants in seven retinal dystrophy-associated genes, *CDHR1*, *CHM*, *CRX*, *ELOVL4*, *PROM1*, *PRPH2*, and *ROM1*, known to phenocopy ABCA4 disease were identified by WES and assessed for enrichment in a discovery cohort of 622 patients with genetically confirmed (≥2 pathogenic variants) Stargardt/ABCA4 disease (n = 622). The control cohort for this analysis consisted of 10,865 healthy individuals from the Institute of Genomic Medicine (IGM) at Columbia University Medical Center (CUMC). Enrichment of a common haplotype at the C-terminal end of *PRPH2* was assessed using allele frequencies derived from an ethnically matched control cohort of 43,029 individuals from the gnomAD database. This analysis was independently validated in a replication cohort 408 of patients from the Baylor College of Medicine (BCM) and the National Eye Institute (NEI).

The overall design of the study is given in **Fig 1**. We searched for putative *trans*-modifiers in patients with STGD1/ABCA4 disease by performing the association analyses in a discovery cohort of 622 bi-allelic, confirmed cases from the Columbia University center. This cohort included 79% patients of European decent; the determination of ethnic composition of the patient cohort and the ethnically matched control cohorts are given in Methods. To confirm some of these findings, we used a replication cohort of 408 bi-allelic cases obtained from two centers, Baylor College of Medicine and the National Eye Institute. In addition to the analysis in the entire cohorts, we also divided ABCA4 disease patients into three genetically defined groups. The first group included cases with the causal, hypomorphic p.Asn1868Ile variant (83 and 60 patients, respectively). The second group was defined as cases with the causal p.Gly1961Glu variant (153 and 70 patients). The third group included all other bi-allelic ABCA4 disease cases (386 and 278 patients). This group also includes all “other” p.Asn1868Ile cases, where the hypomorphic variant is not causal, but rather in *cis* with a known pathogenic variant. We compared the frequencies of rare variants in coding and splice-site sequences (as derived from WES) in 622 cases to the ethnically matched control cohort of 10,865 individuals from Columbia Institute for Genomic Medicine (IGM) database that we had used before in studies of ABCA4 disease [22,24]. The qualifying variants were defined by two filters, the minor allele frequency (MAF) and the *in silico* functional filter, combined annotation dependent depletion (CADD) score >25. For common variants, the allele frequencies in the general population, at exactly the same fractions of ethnicities as in cases, were derived from the gnom AD database (see Methods). The primary initial goal of the study was to test the hypothesis that rare and/or common variants in the 7 macular disease-associated genes are statistically significantly associated with ABCA4 disease cases in general and, for common variants, in the two genetically determined subgroups. The next question after obtaining these results was if the identified variants are affecting disease penetrance or disease expression.

### Analysis of rare variants in seven genes causing STGD1-like macular dystrophies

Analysis of the cohort of 622 bi-allelic ABCA4 disease cases revealed a moderately elevated occurrence of variants in *CDHR1*, *CHM*, *CRX*, *ELOVL4*, *PROM1*, *PRPH2*, and *ROM1* genes in the patient group compared to the control cohort (**Table 1**). Specifically, while there was no difference in very rare (MAF<0.00001) variants between cases and controls after applying both MAF and CADD>25 filters, the increasing ratio of variants in cases vs controls became evident with increasing MAFs (**Table 1**). In these analyses, 3.9% of patients vs 2.8% of control individuals (p = 0.13) harbored at least one variant in the 7 genes with MAF<0.005 and CADD>25, the latter filter suggesting a functional effect. However, none of these differences in carrier proportions reached statistical significance.

**Table 1:** Heterozygous individuals in ABCA4 disease and control cohort harboring rare variants in macular dystrophy genes *CDHR1*, *CHM*, *CRX*, *ELOVL4*, *PROM1*, *PRPH2*, *ROM1*. All comparisons were performed with the two-sided Fisher’s Exact Test (FET). MAF, minor allele frequency; gnomAD, genome aggregation database; CADD, combined annotation dependent depletion; nd, not detected.

MAF + CADD>25	ABCA4 disease patients (622)	Controls (10,865)	Unadjusted p-value
<0.005	24 (3.9%)	306 (2.8%)	0.1302
<0.001	15 (2.4%)	186 (1.7%)	0.1956
<0.0001	10 (1.6%)	105 (1.0%)	0.1182
<0.00001	2 (0.3%)	36 (0.3%)	1
gnomAD nd	2 (0.3%)	35 (0.3%)	1

**Fig 2:**
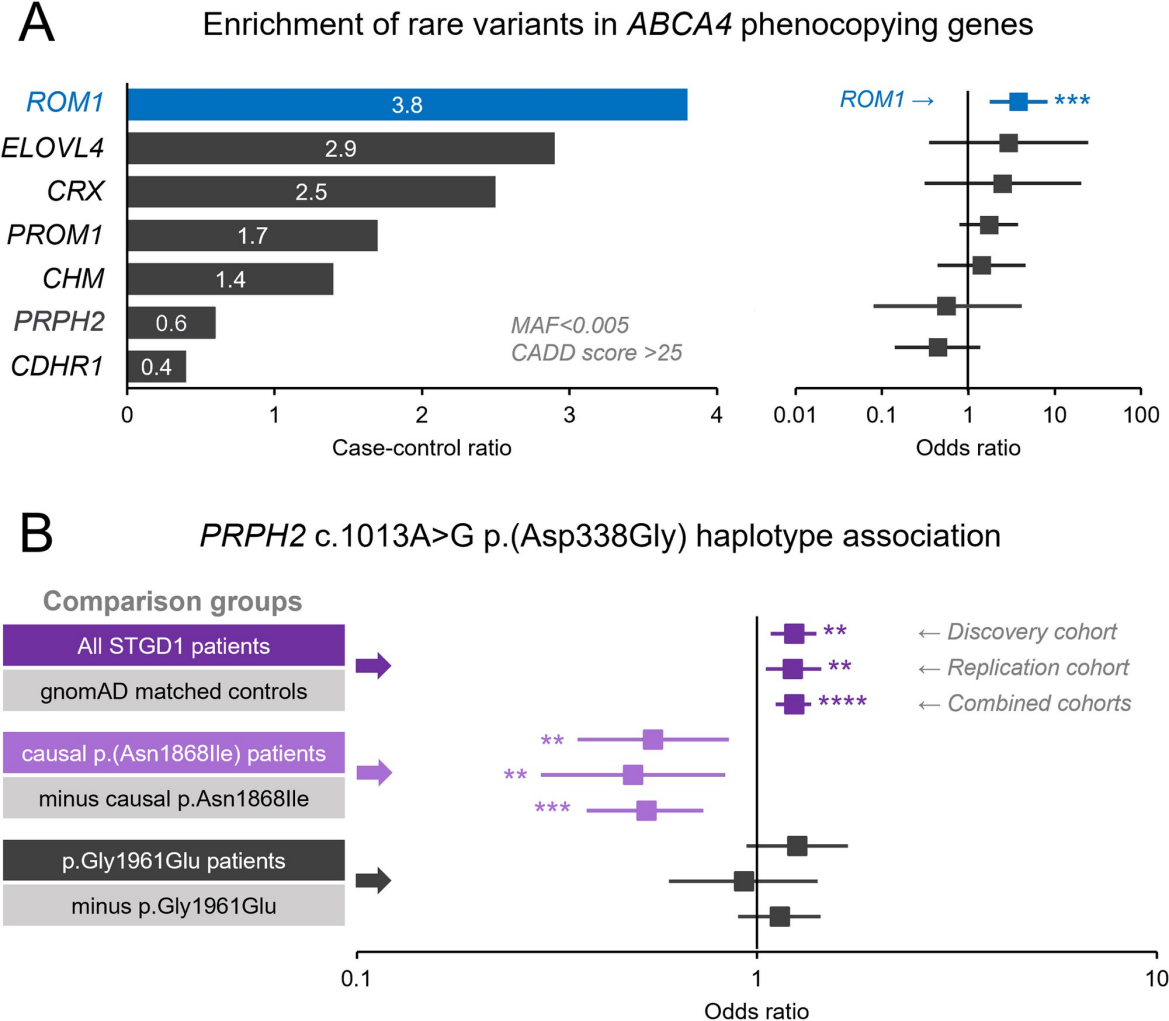
Case-control analysis of rare and common variants of seven macular dystrophy genes in the Stargardt/ABCA4 disease. (A) Moderately rare variants in *ROM1* with a minor allele frequency (MAF) < 0.005 and CADD (v1.3) score >25 were significantly enriched in the discovery cohort compared to a matched control cohort by a factor of 3.8 (horizontal blue bar). The case-control ratio is the percentage of cases harboring qualified variants among the ABCA4 disease cohort (n = 622) divided by the percentage of cases harboring qualified variants matched controls (n = 10,865). (B) Forest plot of odds ratios with 95% confidence intervals (whiskers) of variant enrichment between patients and controls. OR > 1 signifies enrichment in ABCA4 disease. (B) The minor c.1013A-tagged haplotype in the C-terminal end of *PRPH2* was significantly elevated across all patients in the discovery, replication, and combined cohorts compared to matched general population frequencies reported in gnomAD. Analysis of genetically determined clinical subgroups suggested a significant reduction of the same haplotype in patients harboring the hypomorphic p.Asn1868Ile allele. For significance level, two asterisks (**) denotes P ≤ 0.01, three asterisks (***) denotes P ≤ 0.001. and four asterisks (****) denotes P ≤ 0.0001.

We did not find a statistically significant enrichment of cumulative rare coding variants in the seven STGD1-like macular dystrophy genes in our patient cohort with ABCA4 disease without or with the Bonferroni-corrected significance threshold of 0.05/5 = 0.01. However, when we analyzed the 7 genes individually, there was a statistically significant enrichment of variants in one of the 7 genes, *ROM1* (**Table 2, Fig 2**). Eight (1,3%) of 622 patients harbored a variant with MAF<0.005 and CADD>25 filters compared to 37 (0.3%) of 10,865 controls (p = 0.00024; OR 3.81 95% CI [1.77; 8.22]). The association was statistically significant also after Bonferroni correction for 7 comparisons (7 genes), resulting in the significance threshold of 0.05/7 = 0.007 (**Tables 2 and** S1). While the frequency of rare *ROM1* alleles is ~4x higher in cases with ABCA4 disease than in matched controls, we did not notice any discernible differences in phenotypes of these 8 cases. Moreover, the number of carriers of possibly functional *ROM1* alleles is very small (8), so any further separation into *ABCA4* genotype or phenotype group will have no statistical power. Therefore, the observed association has to be studied in much larger cohorts of ABCA4 disease, which is only possible in meta-analyses of data acquired in many centers including a sufficient number of ABCA4 disease cases.

**Table 2:** Heterozygous individuals in ABCA4 disease and control cohort harboring variants with MAF<0.005 and CADD score >25, in each macular dystrophy gene *CDHR1*, *CHM*, *CRX*, *ELOVL4*, *PROM1*, *PRPH2*, and *ROM1*. All comparisons were performed with the two-sided FET.

Gene	ABCA4 disease patients (622)	Controls (10,865)	patients/controls (%/%)	Unadjusted p-value	OR
*CDHR1*	3 (0.5%)	119 (1.1%)	0.4	0.147	0.44 [0.14; 1.38]
*CHM*	3 (0.5%)	37 (0.3%)	1.4	0.56	1.42 [0.44; 4.61]
*CRX*	1 (0.2%)	7 (0.1%)	2.5	0.376	2.49 [0.31; 20.33]
*ELOVL4*	1 (2%)	6 (0.1%)	2.9	0.3	2.91 [0.35; 24.25]
*PROM1*	7 (1.1%)	71 (0.7%)	1.7	0.163	1.73 [0.79; 3.78]
*PRPH2*	1 (0.2%)	31 (0.3%)	0.6	0.567	0.56 [0.08; 4.13]
*ROM1*	8 (1.3%)	37 (0.3%)	3.8	0.000241	3.81 [1.77; 8.22]

While most of the nonsynonymous variants in *CDHR1*, *CHM*, *CRX*, *ELOVL4*, *PROM1*, *PRPH2*, and *ROM1* coding regions identified in patients with ABCA4 disease are moderately to very rare (under the MAF<0.005 threshold), some variants present with higher MAFs. Frequencies of these variants are in some cases also significantly different between ethnic groups (**S2 Table**). The allele frequencies, as presented in all genetic databases for the most frequent variants, such as *ROM1* c.353G>C (p.Gly118Ala), and *PRPH2* c.910C>G (p.Gln304Glu), c.929G>A (p.Arg310Lys), and c.1013A>G (p.Asp338Gly), represent, in fact, the major alleles, since the human reference genome is harboring respective minor allele nucleotide, and variant calling reports differences from the reference sequence. Based on the actual minor allele frequencies, the *PRPH2* variants c.910C (p.Gln304) and c.1013A (p.Asp338) are the most common variants in the STGD1-like macular dystrophy genes at MAF~0.2 each in the European population (**S2 Table**).

### Analysis of frequent variants and the *PRPH2* haplotype

The two *PRPH2* variants, c.910C (p.Gln304) and c.1013A (p.Asp338), are in almost complete LD and tag a haplotype, which is present in ~20% of the alleles of the European general population and in ~21% of the alleles of our ethnically matched control cohorts (Methods and **Tables 3 and 4**). Half of these cases also harbor the c.929G (p.Arg310) variant (MAF~0.1) and the three SNPs form the six possible haplotypes as shown in **Table 3**.

**Table 3:** Haplotype frequencies in the 3’ end of the *PRPH2* gene in ABCA4 disease cohorts and genetically determined disease subgroups. For *PRPH2*, nucleotide positions and protein translation correspond to CCDS4871.1 and NP_000313.2, respectively; for *ABCA4*, CCDS747.1 and NP_000341.2, respectively. Nucleotide numbering uses the A of the ATG translation initiation start site as nucleotide 1. The haplotype frequencies were determined directly from sequence data.

*PRPH2* haplotypes		Discovery cohort (allele count and (%))			Replication cohort (allele count and (%))		
cDNA positions 910, 929, 1013	amino acids 304, 310, 338	all patients (n = 622)	causal p.Asn1868Ile cases (n = 83)	p.Gly1961Glu cases (n = 153)	all patients (n = 408)	causal p.Asn1868Ile cases (n = 60)	p.Gly1961Glu cases (n = 70)
**G-A-G**	**EKG**	937 (75.3)	139 (83.7)	220 (71.9)	613 (75.1)	102 (85)	107 (76.4)
**C-G-A**	**QRD**	136 (10.9)	14 (8.4)	52 (17)	99 (12.1)	8 (6.7)	23 (16.4)
**C-A-A**	**QKD**	151 (12.1)	13 (7.8)	34 (11.1)	95 (11.6)	10 (8.3)	10 (7.1)
**G-A-A**	**EKD**	19 (1.5)	0	0	6 (0.7)	0	0
**G-G-A**	**ERD**	0	0	0	2 (0.2)	0	0
**C-A-G**	**QKG**	1 (0.08)	0	0	1 (0.1)	0	0

**Table 4:** *PRPH2* c.1013A>G p.(Asp338Gly) genotype and allele frequency comparisons in ABCA4 disease cohorts and genetically determined disease subgroups. For *PRPH2*, nucleotide positions and protein translation correspond to CCDS4871.1 and NP_000313.2, respectively; for *ABCA4*, CCDS747.1 and NP_000341.2, respectively. Nucleotide numbering uses the A of the ATG translation initiation start site as nucleotide 1. All comparisons were performed with the two-sided FET. P, uncorrected p-values. Bonferroni correction resulted in the significance thresholds of p = 0.05/4 = 0.0125 for the Discovery cohort and p = 0.05/3 = 0.017 for the Replication and Combined cohorts. AF, allele frequency; P, unadjusted p-value; gnomAD, genome aggregation database. *Replication cohort did not include the data for the c.4253+43G>A variant.

	Individuals	Genotypes (%)			AF	P	OR 95% CI []
Discovery cohort		AA	AG	GG			
all STGD1 patients	622	5.5	38.6	55.9	0.75	0.0014	1.24 [1.085; 1.406]
vs gnomAD matched controls	43029	4.5	33.1	62.4	0.79		
causal p.Asn1868Ile patients	83	0	32.5	67.5	0.84	0.0065	0.55 [0.357; 0.85]
vs minus causal p.Asn1868Ile	539	6.3	39.5	54.2	0.74		
p.Gly1961Glu patients	153	7.8	40.5	51.6	0.72	0.1184	1.26 [0.942; 1.687]
vs minus p.Gly1961Glu	469	4.7	38	57.3	0.76		
causal p.Asn1868Ile and c.4253+43G>A*	97	0	29.9	70.1	0.85	0.00057	0.49 [0.32; 0.738]
vs minus causal p.Asn1868Ile and c.4253+43G>A	525	6.5	40.2	53.3	0.73		
**Replication cohort**							
all STGD1 patients	408	7.3	34.8	57.8	0.75	0.0095	1.23 [1.052; 1.449]
vs gnomAD matched controls	43029	4.5	33.1	62.4	0.79		
causal p.Asn1868Ile patients	60	0	30	70	0.85	0.0073	0.49 [0.289; 0.833]
vs minus causal p.Asn1868Ile	348	8.6	35.6	55.7	0.74		
p.Gly1961Glu patients	70	7.1	32.9	60	0.76	0.7215	0.93 [0.603; 1.419]
vs minus p.Gly1961Glu	338	7.4	35.2	57.4	0.75		
**Combined cohorts**							
all STGD1 patients	1030	6.2	37.1	56.7	0.75	4.00E-05	1.24 [1.116; 1.367]
vs gnomAD matched controls	43029	4.5	33.1	62.4	0.79		
causal p.Asn1868Ile patients	143	0	31.5	68.5	0.84	0.00014	0.53 [0.376; 0.735]
vs minus causal p.Asn1868Ile	887	7.2	38	54.8	0.74		
p.Gly1961Glu patients	223	7.6	38.1	54.3	0.73	0.2874	1.14 [0.897; 1.445]
vs minus p.Gly1961Glu	807	5.8	36.8	57.4	0.76		

We defined the c.1013A (p.Asp338) variant as the haplotype-tagging SNP in further analyses and compared the frequency of this haplotype in 3 separate cohorts as follows (**Table 4**). In the Discovery cohort, the c.1013A (p.Asp338) minor allele was significantly increased in the entire ABCA4 disease patient cohort (622 cases) as compared to the ethnically matched general population; MAF 0.25 vs 0.21 (p = 0.0014) (**Table 4**). We also performed the analysis in genetically determined subgroups and observed statistically significant differences in MAFs between these when compared to the rest of patients. In the hypomorphic subgroup of the ABCA4 disease, including cases with the p.Asn1868Ile and c.4253+43G>A variants, the allele frequency for the c.1013A (p.Asp338) variant was significantly lower, 0.15 in 97 patients vs 0.27 in the remaining 525 Stargardt patients (p = 0.00057; OR 0.49 95% CI [0.32; 0.74]) (**Discovery cohort, Table 4**). Importantly, the genotype analysis revealed a complete absence of the c.1013A (p.Asp338) minor allele homozygotes in the hypomorphic patient group (**Table 4**) compared to 12/153 (7.8%, p = 0.00038) in the patient group defined by the p.Gly1961Glu mutation, and 21/372 (5.6%, p = 0.0015), in the remaining ABCA4 disease patients. The association was also significant when the p.Asn1868Ile cases were analyzed separately (p = 0.0065), but not significant for the patient group defined by the p.Gly1961Glu mutation, p = 0.1184 (**Table 4**). This group showed an opposite trend in allele frequencies, presenting with two times higher MAFs for c.1013A (p.Asp338) compared to the hypomorph group (0.28 vs 0.15, p = 0.00066). The observed differences remained statistically significant also after Bonferroni correction for 4 comparisons was applied, resulting in the significance threshold of 0.05/4 = 0.0125 (**Discovery cohort, Table 4**).

To confirm and validate these associations, we performed 3 of the same analyses in an independent Replication cohort consisting of 408 bi-allelic ABCA4 disease patients from Baylor College of Medicine (BCM) and the National Eye Institute (NEI). The minor haplotype tagged by the c.1013A (p.Asp338) allele was, again, significantly increased in the entire replication cohort as well when compared to the matched control cohort, 0.25, p = 0.0095) (**Table 4**), confirming the statistically significant association observed in the Discovery cohort. The *PRPH2* haplotype frequencies were similar in both, Discovery and Replication cohorts, suggesting close matching by ethnicity in the two cohorts. Importantly, this was true for the entire cohorts and for defined disease subgroups (**Tables 3 and 4**). For the hypomorphic disease group, the fraction of major haplotype harboring the more frequent variants in *PRPH2* cDNA positions 910, 929, and 1023 –G-A-G corresponding to p.[Glu304;Lys310;Gly338] (EKG), was increased, compared to the rest of patients, 84% vs 74% (p = 0.0065) in the CU cohort and 85% vs 74% (p = 0.0073) in the replication cohort (**Table 4**). We did not identify minor allele homozygotes in either of the cohorts in hypomorphic disease group, compared to 4% (p = 0.00066) in the general population, and 6.2% in the rest of the ABCA4 disease patients (p = 3.3E-5). The less frequent haplotypes C-G-A for p.[Gln304;Arg310;Asp338] (QRD) and C-A-A for p.[Gln304;Lys310;Asp338] (QKD) combined were increased in the p.Gly1961Glu patient group, 28.1% compared to 16.2% in the hypomorph group in CU cohort, and 23.5% vs 15%, respectively, in the replication cohort. The remaining haplotypes were rare (~1–2%) (**Table 3**).

When combining the discovery and replication cohorts (Combined cohorts, **Table 4**), totaling 1030 patients with ABCA4 disease, the association with the *PRPH2* haplotype-tagging c.1013A (p.Asp338) allele is statistically very strong, p = 4.00E-05, OR 1.24 95% CI [1.12; 1.37] for all patients and p = 0.00014, OR 0.53, 95% CI [0.38, 0.74] for the hypomorph subgroup (**Table 4**) and remains significant after Bonferroni correction for 3 comparisons (significance threshold p = 0.05/3 = 0.017). In summary, the combined analyses identified the frequent haplotype in the 3’ end of the *PRPH*2 gene, consisting of c.910C>G (p.Gln304Glu), c.929G>A (p.Arg310Lys), and c.1013A>G (p.Asp338Gly) variants, as statistically significantly associated with ABCA4 disease in general and with the specific, hypomorphic genotype subgroup in particular. We identified a ~12% shift toward the major G-A-G (EKG) haplotype in the disease group defined by hypomorphic p.Asn1868Ile and c.4253+43G>A variants and, consequently, a reduced number of cases harboring the C-G-A (QRD) and C-A-A (QKD) haplotypes in this disease subgroup (**Table 3**). The absence of minor allele homozygotes in the hypomorphic disease group is the main driver behind the increased prevalence of the major, c.1013G (p.Gly338) allele in this patient group.

## Discussion

Here we describe the first genetic *trans*-modifiers for ABCA4 disease consisting of rare and common variants outside of the *ABCA4* locus that are associated with the STGD1/ABCA4 disease in general, and with a specific, genetically defined, subgroup. *ABCA4*-associated disease phenotypes are extremely heterogeneous and, in addition to the >1,500 disease-causing variants identified in the *ABCA4* locus thus far, these are influenced by genetic modifiers. We have described *cis*-modifiers in the *ABCA4* locus previously [13–15]. Similarly, rare cases of causal variants in two genes together influencing the phenotype have been identified [20,25]. However, while their presence was anticipated due to overlapping phenotypes of macular dystrophies and plausible functional interaction of proteins harboring causal variants, until now the putative effect of genetic *trans*-modifiers has remained elusive.

From both the clinical and functional/biological perspective, the most likely gene to *trans*-modify ABCA4 disease is *PRPH2*. First, a significant fraction of disease phenotypes caused by *PRPH2* variants are clinically indistinguishable from ABCA4 disease [18,22]. At least 10% of the disease cases mimicking ABCA4/STGD1 are caused by *PRPH2* variants [22]. Second, PRPH2 and ABCA4 co-localize in the same subcellular region, the rims of photoreceptor (PR) outer segment (OS) disc membranes [26,27]. The proteins perform very different functions. ABCA4 is the N-ret-PE transporter in the visual cycle [28] and PRPH2, often in complex with ROM1, is a structural protein necessary to form the PR disc loops to accommodate the large ABCA4 protein, which otherwise does not fit into the densely packed PR OS disc membranes [29,30]. Given both the structural and physiological similarity between ROM1 and PRPH2, our finding that variants in these two genes, *PRPH2* and *ROM1*, modify ABCA4 disease is, therefore, not unexpected. However, the exact mechanism of this modifying allelic interaction remains elusive. While the ABCA4 and PRPH2/ROM1 proteins are co-localized to PR outer segment disc rims, their physical interaction, while highly likely, has not been demonstrated. Most recently, the high-definition structure of ABCA4 protein was determined by cryo-EM [31] but not for PRPH2 nor ROM1, or their complexes. Moreover, the PR OS membrane structure, which could reveal the functional effect through protein-protein interactions or conformational changes to the membrane, has yet to be determined.

The function of the PRPH2 C-terminus, which also contains the trans-associated G-A-G (EKG) haplotype, has been extensively studied [32]. It is considered a critical functional domain performing several crucial roles, including regulating membrane curvature and fusion, protein trafficking, and ectosome secretion. Since the PRPH2 C-terminal function includes initiating PR OS morphogenesis and sensing or regulating membrane curvature, it is crucial for properly formed PR OS disc rims, which is where ABCA4 functions as the flippase of vitamin A derivatives, 11-*cis* and all-*trans* retinal in the complex with phosphatidylethanolamine (PE), N-ret-PE [28,33]. It is therefore plausible to hypothesize that even common, non-causal *PRPH2* variants influence the ABCA4 function. Additional evidence for the functional effect of the common haplotype in the C-terminal end, G-A-G (EKG), was recently provided by *in silico* modeling of the common variants c.910C>G (p.Gln304Glu), c.929G>A (p.Arg310Lys), and c.1013A>G (p.Asp338Gly)(34). Specifically, the c.1013G (p.Gly338) variant, and the resulting G-A-G haplotype, was predicted to affect the protein function the most by destabilizing the C-terminus of PRPH2 by affecting the protein folding. The authors concluded that the G-A-G (EKG) haplotype will lead to a possible alteration of PRPH2 binding with melanoregulin and other outer segment proteins, followed by photoreceptor outer segment instability [34]. While it is unlikely that a major haplotype present in ~80% of the general population would have a significant detrimental effect on protein function, the analysis does suggest that this haplotype and specifically the c.1013G (p.Gly338) variant may have an incremental but, nevertheless, modifying pathogenic effect. Importantly, another study discussing phenotype differences in PRPH2 disease, provides even more supportive evidence for the role of the G-A-G (EKG) haplotype in disease expression [35]. In 19 families, where the disease segregated with the dominant *PRPH2* c.828+3A>T variant, the phenotypes were very discordant, ranging from mild pattern dystrophy to severe cone-rod dystrophy, retinitis pigmentosa and central areolar chorioretinal dystrophy. Comparison of the in *trans* 3’ haplotype frequencies in the mild and severe groups revealed that the G-A-G (EKG) haplotype was associated with the severe group while the C-A-A (QKD) haplotype was exclusively present in the mild group [35], again suggesting that the G-A-G (EKG) haplotype is “more severe”. All of the above provides direct supportive evidence for our results–the more severe G-A-G (EKG) haplotype is statistically significantly elevated among patients with mild disease caused by *ABCA4* hypomorphic alleles, while lower, relative to the general population, in all other ABCA4 disease patients. Therefore, our data suggest that haplotypes in the C-terminal end of the PRPH2 protein also have a functional effect in *trans* as modifiers of ABCA4 disease. The likely main functional/pathogenic effect is derived from the significantly increased major c.1013G (p.Gly338) allele frequency in the hypomorph group. The hypomorph group includes patients with late-onset, mild disease defined by the p.Asn1868Ile allele with low penetrance [16,36,37]. Therefore, addition of a risk-enhancing *trans*-modifier allele likely increases the disease incidence for this specific subgroup of ABCA4 disease.

The role of *PRPH2* variation in dominant and recessive retinal diseases have been extensively studied, however, much less is known about its oligomerization partner, retinal outer segment membrane protein 1 (ROM1, OMIM #180721). ROM1 is a photoreceptor-specific integral membrane protein also localized to the rim regions of rod and cone outer segments [38–40], where it oligomerizes with PRPH2 through both covalent and noncovalent interactions [41]. However, while ROM1 and PRPH2 share 35% amino acid identity, their functional roles are clearly different. Although *Rom1*^−/−^ mice exhibit a much milder phenotype than *Prph2*^*-/-*^, ROM1 has been shown to play a significant role in OS biogenesis, especially in the maturation of the OS [41,42]. Unlike *PRPH2*, where >160 disease-causing variants have been identified resulting in a spectrum of autosomal recessive and dominant retinal degenerations [43–45], there have been only two reports of *ROM1* variants being causal in a disease phenotype. First, a digenic inheritance with *PRPH2* was described in 1997 [46] and only recently a homozygous frameshift *ROM1* variant was identified as causal in a late-onset pattern dystrophy [23]. Therefore, while likely pathogenic, including definite loss-of-function, variants are not rare in *ROM1*, they almost never directly result in a disease phenotype. However, by utilizing mouse models, it was recently shown that *ROM1* contributes to phenotypic variability of PRPH2 disease, more importantly, to the phenotype of the disease caused by specific *PRPH2* variants [47]. These data further corroborate our findings that specific, genetically and phenotypically defined, subgroups of ABCA4 disease are modified by both *ROM1* and *PRPH2* alleles.

In summary, we identified rare variants in the *ROM1* gene and a common haplotype in the *PRPH2* gene that are associated with ABCA4 disease. Specifically, the strongest association is with the major *PRPH2* haplotype, which is decreased by ~4% in ABCA4 disease overall and by ~7% in the p.Gly1961Glu subgroup. At the same time, this haplotype is increased by 12% in the genetically determined sub-cohort defined by the p.Asn1868Ile variant. The p.Asn1868Ile variant, at a MAF~0.07 in populations of European descent, is a hypomorphic allele with limited penetrance [13,16]. We and others have defined conditions under which the variant becomes clinically pathogenic. It requires a deleterious variant on the allele in *trans* and or other *cis*-modifiers to become fully penetrant [13,48]. In the current study, we have controlled for all known *cis*-modifiers for the p.Asn1868Ile-defined group in all cohorts so that it includes only “pure” p.Asn1868Ile allele cases. All other cases with the variant are included in the rest of the cohort. Since the *PRPH2* major, “risk” haplotype is significantly increased in the p.Asn1868Ile cohort as compared to the rest of cases (and even to the general population) we suggest that the presence of this haplotype further increases the penetrance of the “pure” p.Asn1868Ile allele. Despite the strong association, the consequence of this *trans*-modifying event is not discernible in an individual, as opposed to known *cis*-modifiers, that are associated with both increased penetrance and often drastically different phenotypic outcomes. We suggest that the main reason is that the known *cis*-modifiers directly affect the ABCA4 function, while the effects of variation in *PRPH2/ROM1* on ABCA4 function (and possibly vice versa) may indeed be indirect and nonspecific. Rather, variable defects in PR OS membrane structure resulting from moderately dysfunctional PRPH2/ROM1 may have an incremental but cumulatively negative effect on the ABCA4 disease process. Such nonspecific events would likely not manifest significantly at the clinical level but rather, as we hypothesize in this case, contribute to overall disease penetrance, particularly with hypomorphic genotypes. Therefore, our findings should caution against the generally held expectation that *trans*-modifiers of Mendelian diseases have to be functionally coupled to the underlying causal gene; i.e., forming an “interactome” [1].

The implications of this study should be further explored on various levels. Similar analyses of other independent patient and control cohorts would lay a groundwork for larger meta-analyses of individual disease subgroups with sufficient statistical power. Mechanistic studies on possible protein interactions, or PR OS membrane modifications, involving ABCA4 and the PRPH2/ROM1 complex, would also be informative.

## Conclusion

Extensive genetic and clinical heterogeneity of ABCA4 disease, incomplete penetrance of many alleles, and the presence of functionally unrelated phenocopying genes have suggested a role for additional non-allelic factors in disease expression [8]. By describing *trans*-modifiers in a significant fraction of Stargardt/ABCA4 disease, this study adds to the explanation of an unusually complex genetic background. Specifically, this study establishes a clear association between *ABCA4* and *PRPH2/ROM1* variation in disease. While the individual functions of ABCA4 and PRPH2/ROM1 proteins are known not to be directly related, both share a common subcellular milieu and underlie very similar disease outcomes in patients. As such, incremental dysfunction in one may affect the other in an indirect but additive manner. The precise effect(s) remain to be understood mechanistically; however, more importantly, these findings should broaden the manner in which genetic modifiers are defined and sought after in both ABCA4 disease and other Mendelian disorders and suggest continuing and expanding searching for both *cis-* and *trans-*modifying alleles to better elucidate and explain precise biological effects and clinical consequences in ABCA4 disease.

## Methods

### Ethics statement

All patients from Columbia University and The Pangere Center for Inherited Retinal Diseases were consented in writing under the protocols #AAAI9906 approved by the Institutional Review Board at Columbia. The patients from the National Eye Institute were enrolled with written informed consents under two protocols, NEI NCT02471287, approved by the Institutional Review Board and the eyeGENE Program (NEI NCT00378742). The control samples from Institute of Genomic Medicine and patient samples from Baylor College of Medicine were collected with written informed consent at the time of recruitment and approved for research by site-specific institutional review boards and ethics committees. All study procedures adhered to the tenets established in the Declaration of Helsinki.

### Study subjects

The 622 Stargardt patients of mostly European descent in the discovery cohort were recruited and clinically examined during a 20-year period at Columbia University, and The Pangere Center for Inherited Retinal Diseases, The Chicago Lighthouse. Prior to study enrollment, all patients underwent complete ophthalmic examinations by retinal specialists, which included a dilated slit-lamp examination, assessment of best-corrected visual acuity, thorough family histories, retina imaging and full-field electroretinogram (ffERG) testing to aid in clinical diagnoses. All study subjects were confirmed to carry bi-allelic *ABCA4* disease-causing variants identified in previous sequencing of the *ABCA4* coding sequences or entire genomic locus using the Illumina TruSeq Custom Amplicon protocol (Illumina, San Diego, CA) or single-molecule molecular inversion probes (smMIPs) [49]. The race and ethnic composition of the study cohort (**S1 Fig**) was determined based on both self-reported responses from each individual and sequencing-based clustering analyses. All study subjects completed a questionnaire that included inquiries about their maternal and paternal descent, allowing to determine patient’s ethnicity. To further account for ethnic heterogeneity in both the ABCA4 disease case and control cohorts, we applied the Louvain method of community detection on the first six principal components to extract genetically determined clusters reflecting geographic ancestry. Using a neural network pre-trained on a dataset of ethnically confirmed samples, we identified probability estimates for the following ethnicity subpopulations, (1) African, (2) East Asian, (3) European, (3) Latino, (4) Middle Eastern, and (5) South Asian, which were assigned to qualifying cases for whom the probability was greater than 95%. Cases with probability estimates below the 95% cut off were designated as “Admixed.” We then performed further dimensionality reduction by uniform manifold approximation and projection (UMAP) on the first 6 principal components to determine the relationship between cluster memberships assigned by the neural network.

The control cohort of 10,865 individuals was selected from other Institute for Genomic Medicine (IGM) studies [22,24]. Controls were known to not have ophthalmic disease, liver disease, kidney disease, metabolic disease, or ALS. The same clustering analysis as for the case cohort was applied to the IGM control cohort and the exact fractions of controls of each ethnicity were included in the resulting 10,865 control cohort prior to any association analyses. The gnomAD-derived control cohort of 43,029 individuals was derived by combining the *PRPH2* allele frequencies in the same proportions of ethnicities as in cases. These analyses allowed us close to perfect matching of case and control cohorts.

The replication cohort included 408 Stargardt patients from two centers, Baylor College of Medicine and the National Eye Institute. The 135 patients recruited at Baylor College of Medicine were predominantly of non-Finnish European descent. The patients underwent clinical ophthalmic assessment prior to the enrollment. After recruitment, the subjects underwent gene panel sequencing and/or whole exome sequencing (WES).

The 273 patients from the National Eye Institute were clinically evaluated for the presence of Stargardt/ABCA4 disease and underwent genetic screening through various diagnostic laboratories. Further details of diagnostic screenings are provided below.

### Sequencing and analysis

At Columbia University, exome capture kits v.5 and v.7 from Agilent Technologies (Santa Clara, CA) and Integrated DNA Technologies’ (Coralville, IA) Exome Research Panel v.1 were used for whole exome sequencing of all study subjects and the control cohort. All exome data of the discovery cohort were processed using the IGM alignment and annotation pipeline for standardized analysis outcome. Samples were sequenced on Illumina next-generation sequencing machines using DRAGEN Bio-IT Platform v.2.5.1 (Illumina) to align reads to the Genome Reference Consortium Human Build37, calling variants in accordance with the Genome Analysis Tool Kit (GATK, v.4.0.2.1) Best Practices Workflow, using ATAV (v.7.0.16), an IGM variant-calling pipeline [24].

Variant data for the macular dystrophy genes *CDHR1*, *CHM*, *CRX*, *ELOVL4*, *PRPH2*, *PROM1*, and *ROM1*, were extracted and compared between patients and controls in collapsing analysis on a single gene level and combined as a group of 7 genes. All samples were required to have at least 10X coverage in at least 90% of the CCDS regions. All samples with second-degree or closer kinship, as evaluated with KING (1.4.2), were excluded. Population substructures were corrected using the EIGENSTRAT (6.1.4) pruning algorithm [50]. We only considered sites with equivalent rates of coverage between cases and controls, removing sites in CCDS (hg19, release 20) with an 11% or greater coverage difference between all cases and all controls.

Qualifying variants per sample, and for the entire patient cohort were defined by allele frequency, from ‘ultra-rare’, with minor allele frequency (MAF)< 0.00001 and absent in The Genome Aggregation Database (gnomAD) [51], to ‘rare’, MAF<0.001, and ‘moderately rare’, MAF<0.005 in gnomAD. *In silico* possible pathogenicity assessment of variants by CADD [52] v.1.3 (>25) was applied as a functional filter. All comparisons were performed with the two-sided Fisher’s Exact Test (FET). Bonferroni multiple-test correction was used where appropriate and specifically described in the Results and Table legends. For example, the significance threshold for the analysis of 7 genes individually (Table 2) was p = 0.05/7 = 0.007.

At Baylor College of Medicine for gene panel sequencing, the DNA was captured with the probes targeting the exonic regions and known intronic variants of known retinal disease genes. The Agilent Hybridization and Wash Kits was used to capture, wash and recover the targeted DNA. For WES, the Nimble Gen Seq Cap EZ Hybridization and Wash kit was used to capture DNA. The captured DNA libraries were sequenced on an Illumina Hi Seq 2000 Platform (Illumina) at Baylor College of Medicine’s Human Genome Sequencing Center (Houston, TX, USA). After sequencing, the reads were aligned to the human reference genome (the hg19/GRCh37 assembly) with BWA (v.0.6.1). The base quality recalibration and local realignment were conducted with the Genome Analysis Tool Kit (GATK, v.3.6). Variant calling was performed with Atlas-SNP2 and Atlas-Indel2.

At NEI, the sequencing was outsourced to commercial laboratories where patient samples were subjected to NGS-based gene panels as follows. At Molecular Vision Laboratory (MVL), patients were sequenced on the Stargardt/Macular dystrophy panel including 17 genes (*ABCA4*, *BEST1*, *CDH3*, *DRAM2*, *EFEMP1*, *ELOVL4*, *IMPG1*, *IMPG2*, *PROM1*, *PRPH2*, *RP1L1*, *TIMP3*, *TTLL5*) or on the Vision panel which included 403 genes involved in genetic eye disorders. At Casey Eye Institute (CEI), patients were subjected to Stargardt/Macular dystrophy panel including 10 genes (*ABCA4*, *BEST1*, *CDH3*, *EFEMP1*, *ELOVL4*, *IMPG1*, *IMPG2*, *PROM1*, *PRPH2*, *TIMP3*). Remaining patients were sequenced at Blueprint Genetics (BpG) for 26 macular dystrophy genes (*ABCA4*, *BEST1*, *C1QTNF5*, *CDH3*, *CERKL*, *CNGB3*, *CRB1*, *CRX*, *CTNNA1*, *DRAM2*, *EFEMP1*, *ELOVL4*, *IMPG1*, *IMPG2*, *MFSD8*, *PRDM13*, *PROM1*, *PRPH2*, *RAX2*, *RDH12*, *RDH5*, *RLBP1*, *RP1L1*, *RPGR*, *RS1* and *TIMP3*) or for 325 retinal dystrophy genes.

Along with genetic testing reports, raw data was received from these labs and was re-processed at NEI through an in-house pipeline. Briefly, the reads were aligned against hg19 reference genome sequence by using BWA aligner. Duplicates were marked by using Picard tool and variant calling was done by GATK and/or Free bayes. Variants were annotated by variant effect predictor (VEP) or intervar/annovar and were used to develop a database by using GEMINI. Custom queries were used to query GEMINI database to output all annotated variants in *ABCA4* and *PRPH2* genes and were used for downstream association analysis.

In summary, while different screening methods were used at the 3 centers, all cases in the discovery and replication cohorts had complete sequencing and data analysis performed for the *ABCA4* and *PRPH2* genes. Therefore, the resulting data used for haplotype analysis part were complete and identical in quality. All cases and controls for the rare variant analyses received WES and were annotated and analyzed on the same, Columbia IGM, pipeline.

## Supporting information

S1 Table Moderately rare (MAF<0.005) nonsynonymous variants in *ROM1* coding sequences, identified in STGD1/ABCA4 disease patients (n = 622) and in a control cohort (n = 10,865). (XLSX)

S2 Table Allele frequencies of all coding nonsynonymous variants identified in macular dystrophy genes *CDHR1*, *CHM*, *CRX*, *ELOVL4*, *PRPH2*, *PROM1*, *ROM1*, in 622 STGD1/ABCA4 disease patients. (XLSX)

S1 Fig Ethnic composition of the ABCA4 disease cohort in the study. Race and ethnic backgrounds of each patient in the cohort were derived from both self-reported biparental lineages and ancestral assignments based on probability estimates from sequencing based clustering analyses (see Methods).
(TIF)
