# Supplementary figures and images for: Rare and common variants in ROM1 and PRPH2 genes trans-modify Stargardt/ABCA4 disease
Source: PLoS Genet. 2022 Mar 30;18(3):e1010129. doi: 10.1371/journal.pgen.1010129 (PMC9000055; doi:10.1371/journal.pgen.1010129)

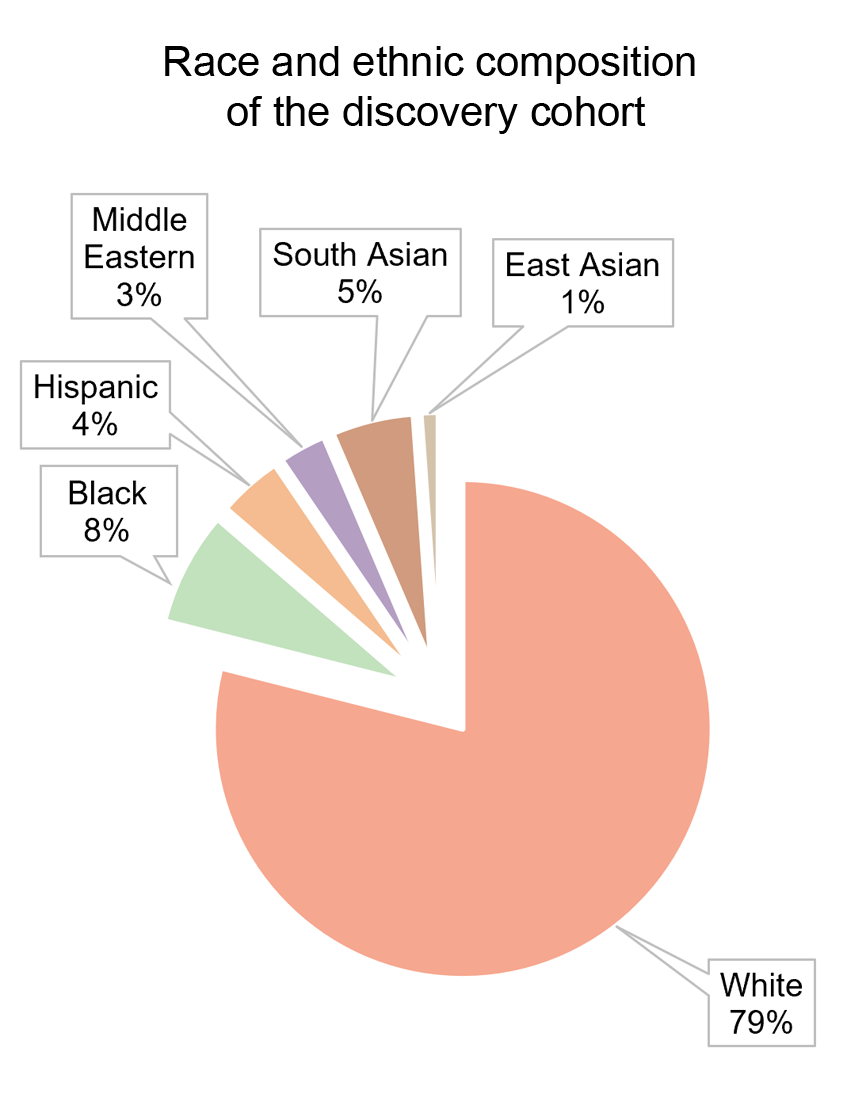

Supplement: S1 Fig — Race and ethnic backgrounds of each patient in the cohort were derived from both self-reported biparental lineages and ancestral assignments based on probability estimates from sequencing based clustering analyses (see Methods). (TIF) [file pgen.1010129.s003.tif]
